# Co-learning commentary: a patient partner perspective in mental health care research

**DOI:** 10.1186/s40900-023-00435-4

**Published:** 2023-04-18

**Authors:** Linda Riches, Lisa Ridgway, Louisa Edwards

**Affiliations:** 1 Patient Partner, Prince George, Canada; 2 Patient Partner, Victoria, Canada; 3 School of Population and Public Health, University of British Columbia (UBC), Vancouver, BC Canada; 4 Centre for Clinical Epidemiology and Evaluation (C2E2), Vancouver Coastal Health Research Institute, 717-828 West 10th Avenue, Research Pavilion, Vancouver, BC V5Z 1M9 Canada

**Keywords:** Depression, Mental health, Patient engagement, Patient involvement, Patient empowerment

## Abstract

**Background:**

Although including patients as full, active members of research teams is becoming more common, there are few accounts about how to do so successfully, and almost none of these are written by patient partners themselves. Three patient partners contributed their lived experience to a three-year, multi-component mental health research project in British Columbia, Canada. As patient partners, we contributed to innovative co-learning in this project, resulting in mutual respect and wide-ranging benefits. To guide future patient partners and researchers seeking patient engagement, we outline the processes that helped our research team ‘get it right’.

**Main body:**

From the outset, we were integrated into components of the project that we chose: thematically coding a rapid review, developing questions and engagement processes for focus groups, and shaping an economic model. Our level of engagement in each component was determined by us. Additionally, we catalyzed the use of surveys to evaluate our engagement and the perceptions of patient engagement from the wider team. At our request, we had a standing place on each monthly meeting agenda. Importantly, we broke new ground when we moved the team from using previously accepted psychiatric terminology that no longer fit the reality of patients’ experiences. We worked diligently with the team to represent the reality that was appropriate for all parties. The approach taken in this project led to meaningful and successfully integrated patient experiences, fostered a shared understanding, which positively impacted team development and cohesion. The resulting ‘lessons learned’ included engaging early, often, and with respect; carving out and creating a safe place, free from stigma; building trust within the research team; drawing on lived experience; co-creating acceptable terminology; and cultivating inclusivity throughout the entire study.

**Conclusion:**

We believe that lived experience can and should go hand-in-hand with research, to ensure study outcomes reflect the knowledge of patients themselves. We were willing to share the truth of our lived experience. We were treated as co-researchers. Successful engagement came from the ‘lessons learned’ that can be used by other teams who wish to engage patient partners in health research.

## Abstract (summary)

Although including patients as members of research teams is becoming more common, there is little information about how to do this successfully. There are even fewer accounts written by patient partners themselves. We argue that successful patient engagement accepts and celebrates the patient partner experience. In this article, we reflect on and share our experiences as patient partners in a Canadian mental health research project. Early on in the project, we were asked to choose which streams of work we would like to work on. In addition, we helped develop surveys about patient engagement in the project. We also had time set aside at each meeting for patient updates. Importantly, we steered the team towards using different mental health terms because they had less stigma and better fit patients’ experiences. We offer the following ‘lessons learned’ about how to engage patients successfully in mental health research, but they are also likely to apply to most health research studies: (1) Engage Early, Often, and with Respect; (2) Carve out and Create a Safe Place; (3) Draw on Lived Experience; (4) Build Trust in the Research Team; (5) Listen, Learn, then Find a New Language; and (6) Cultivate Inclusion Throughout the Project. We believe that the lived experience of patients can and should go hand-in-hand with research. This helps to make sure that the research findings reflect the actual experiences and knowledge of patients. We hope that this article will be a useful guide to other patients and researchers.

## Background

In Canada, there is growing support for patient-oriented health research, nurtured by a unique federal strategy to involve patients, their families and caregivers as meaningful partners in the work [1]. Funded by the Canadian Institutes for Health Research, the national Strategy for Patient Oriented Research [2] has pushed the frontiers of patient engagement for both researchers and the public. However, there are few accounts about how to do patient engagement successfully, and almost none of these are written by patient partners themselves [3]. We are patient partners—or persons with lived experience—on one such research team who can share insights and lessons learned from partnering with researchers in mental health care research.

There are many reasons why it is important, and arguably necessary, to include patients as partners in research related to mental health, including enhancing its relevance and addressing the ethical imperative to do so [4–8]. However, there is the potential to ‘not get it right’. This could include tokenism; from not asking for patient partner input, to asking for opinions, but after a decision has already been made or without ever incorporating any changes. It could also include failing to provide patient partners with adequate information or training, using terminology or acronyms that are not defined or accessible for everyone, or other dismissive behaviours that collectively mean that patient partners are either unable or uncomfortable with contributing to the research process. Not ‘getting it right’ may result in the patient partners and/or the other members of the research team feeling that the challenges are greater than the benefits [9]. Without providing a safe, supportive space, this risk could be magnified for patient partners in mental health research, as a consequence of the ‘emotional labour’—the stress caused by re-telling painful or difficult experiences—of sharing their lived experience of mental illness [10]. The challenges may be fueled by use of jargon, by disrespectful communication or lack of authenticity [8]. Given the risks and challenges, we felt it was important to share, in this article, the processes that helped our research team to ‘get it right’, so that there was mutual respect and undeniable benefit.

We participated in a three-year, multi-component, patient-oriented research project about improving treatments for major depressive disorder in British Columbia, Canada. Based on the successful patient engagement in this project, we have reflected on our experiences as patient partners and would like to share our lessons learned. We hope that this will encourage other patients and researchers to embark on a similar journey together and might provide a counter to some of the reports of tokenistic patient and public engagement [11, 12]. These lessons learned could act as a guide for future projects that want to engage with patient partners, especially those focusing on mental health research.

### Project overview and team composition

The ‘Pharmacogenomics for Depression’ project aimed to evaluate the effectiveness and cost-effectiveness of pharmacogenomic testing for improving antidepressant prescribing. Pharmacogenomics focuses on how a person’s genetics impact their body’s response to medication. It could dramatically improve treatment for major depression by reducing the number of trials it takes to find an effective medication that does not cause intolerable side effects. We wanted to become patient partners on this project because we all have major depression, have struggled for years to find medications that helped us feel better, and we were excited about the possibility that this cutting-edge research could help others avoid going through long and difficult treatment journeys.

The original grant proposal stated that the project would be patient-oriented, and included the provision for “at least two patient partners to be full and active members of our research team”. We were recruited into the study at the ‘collaborate’ level on the spectrum of public engagement [13] as soon as funding began. Although we joined the project after the grant proposal had been written, there was plenty of opportunity to help shape and co-develop the research activities, materials, data interpretation, and dissemination. The project budgeted for and provided a fixed, quarterly financial reimbursement, commensurate with this engagement level in a Canadian context [14].

The 23-member project team included three patient partners, researchers with a range of expertise (e.g., qualitative methods, systematic reviews, mathematical modelling, health economics, administrative data analysis, pharmacogenomics) and clinicians (family physician, genetic counsellors, nurse). There was a range of previous patient engagement experience amongst the patient partners (one first-time, one third-time, one fifth-time patient partner) and the wider research team (just over half had previously worked on a research project with patient partners). However, the project co-leaders, project manager, and knowledge translation lead all had extensive experience with patient-oriented research.

## Lessons learned

### Engage early, often, and with respect

As patient partners, we now know the value of solid project management and oversight. We were involved from the beginning of the research, with patient partner roles specified and reimbursement written into the grant proposal. Trust was established within the first month of the project, and this was probably because we first individually engaged with the project manager (explained about the project, patient partner role, opportunities for involvement, time commitment, answered questions), then had one relationship-building meeting with all three patient partners and the project manager, *and then* attended the first full-team meeting. We were warmly welcomed in the first full-team meeting and, *with advance notice* to allow us to prepare, the team used an effective ice-breaker; i.e., sharing something about one’s heritage, why people wanted to join this project. In the second team meeting, we discussed ‘ways of working’ as a team, including communication preferences, expectations for feedback requests, and how best to share information. This resulted in the full team co-producing a ‘principles of working’ document, which was reviewed at each monthly meeting. In general, suggestions or requests that we made for improving the way information was shared (e.g., PowerPoint slides to be circulated in advance of meetings) were given full consideration and resulted in the team making a change in the way something was done or terminology used. It also helped that team meetings were held each month over Zoom, and so there was frequent connection and opportunity to grow and maintain relationships. Finally, the project manager was in regular contact throughout the project to ensure we felt meaningfully engaged, and offered one-on-one meetings for open discussions 2–3 times per year.

Clarity of workflow of project activities and tasks, effective negotiation and discussion (e.g., on key decisions, language/terminology), and transparency (e.g., clear communication about whether our suggestions were adopted or clear explanations if they could not be addressed due to insufficient evidence or time constraints, etc., which were noted in a decision log) account for much of the projects’ success, for both the patient partners and the researchers. Even something as simple as emails between the project manager and the team was handled so that no one, *especially the patient partners,* felt overwhelmed. For example, email subject lines included the project name, a clear descriptive title, and a respond-by date, if applicable. Complex topics or in-depth discussions were reserved for meetings, while emails were as concise as possible and excluded technical jargon/acronyms, generally were for scheduling purposes or for resolving quick/simple queries, only sent to the specific recipients they concerned, and used different font colour(s) or bolded/highlighted text to draw attention to requests.

### Carve out and create a safe place

All meetings were stigma-free, in which many team members shared personal experiences, and the patient partners felt we could openly disclose our experience with mental illness, without facing or fearing negative judgement. The reciprocal sharing from patient partners and wider research team members helped to contribute to the sense of meetings being a safe place. Practically, the patient partners asked for and received a standing item on the monthly meeting agenda for our questions and comments. A glossary and shared document repository were created at our request, to assist us in understanding the research. The project manager asked us to complete an anonymous survey on our patient engagement experiences in the project; we trusted that the results would benefit the working relationship of the project team, and they have. But most importantly, our privacy was respected with a strong statement in the guiding principles of working together, which the entire research team co-created for this project. These include that *nothing of a personal nature be shared outside of the team, that there is no privilege of perspective/position in this team, and it is safe to say challenging things*. These principles were re-visited at every team meeting.

### Draw on lived experience

Our lived experience of depression has been a foundation of the project meetings, treated with respect and validated by the researchers. We were included in all aspects of the research, clearly seen in our involvement with co-creating the computer simulation model for our project [15], assisting in a literature review, and co-contributing to the creation of interview guides and questionnaires for potential study participants [16]. Since patient partners shaped these study methods, we directly influenced project-wide results. We attended the funders’ Research Oversight Committee meetings and contributed our opinions on the team’s progress through both oral presentations and written summaries in a newly-created section in the funder’s report. The patient partners are the lived experience, and in virtual Zoom interactions, the human face of depression.

### Build trust in the research team

We believe successful patient engagement means trust is established amongst the entire team early on, and it is not necessarily contingent on project length or previous experience. Some of our team had never worked with patients with lived experience before this project. Now, the team is mindful of the reality of treatment and psychosocial factors that can impact treatment for patients, such as financial constraints (medication/psychotherapy affordability, public transport costs to/from appointments, insurance coverage), difficulty getting time off work or childcare coverage for appointments/treatment, stigma, lack of emotional support, courage and willingness to keep switching medications (uncertainty about effectiveness, side effects), motivational challenges when in a depressive episode, etc. By spontaneously sharing about these experiences in meetings, this new awareness has helped forge trust on both fronts. This has been documented in early, middle, and end of project patient engagement surveys. In the patient partner survey, we unanimously reported a high measure of satisfaction. The patient partners asked the researchers to complete a similar series of surveys about their work with patient partners, which further heightened trust. Those survey results, which were anonymized and shared amongst the team, also showed the positive impact of our involvement, such as: “Patient partner feedback has led to questioning, consideration, refinement and shaping of different elements of the project”; “language matters”; “we need to challenge conventions on language use”; and finally, “I will always think twice before using ‘non-adherence’”.

Although trust was established early in this project, trust can also be put to the test at any time. The way these challenges are handled can disrupt or amplify trust. For example, one of the patient partners noted that prevention of death by suicide could result with improved depression treatment, but this had not been considered in the project. The researchers did not know how to respond at first, and the long, awkward pause afterwards left the patient partner worried that maybe they should not have raised this issue. The patient partner and project manager met soon after to discuss the experience and reactions, and then the project leaders decided to raise this topic at the next monthly team meeting: the importance of this issue in mental health, acceptable terminology, how to address this in the project, and a list of resources for support were generated. This ensured we had open, honest discussions about suicide in the project, it was subsequently incorporated into the simulation model, and captured in the results. The way this situation was handled actually strengthened trust even further for us.

### Listen, learn, then find a new language

As patient partners, we were motivated to understand the research better and we took the time to learn acronyms and language connected to the project. We asked for clarification or definitions of unknown terms, reviewed the glossary the project team maintained, and read several articles that the team sent around. Likewise, the researchers have listened to us and adopted less stigmatizing language. For example, after an article [17] was circulated amongst the entire team early into this project, a full-team discussion was held and then a vote was taken to use the term ‘*medication concordance’,* instead of ‘*medication adherence’.* Likewise, we asked the team to refer to ‘*refractory depression’,* instead of ‘*treatment-resistant depression’*. The wider research team created and maintained a list of current terms, alternate possible terms, the preferred terms (by voting), and recorded notes about why this was important. In this way, the project co-created a vocabulary of its own to respect patient partner input.

### Cultivate inclusivity throughout the project

Our participation in this research project on mental health has led to creativity, inclusivity, and a cooperative ethos within our team, with plenty of space for kindness and laughter along the way. We were integrated into components of the project that we chose: thematically coding a rapid review, developing questions and advising on engagement processes for focus groups [16], shaping an economic model [15]. We were offered and accepted invitations for contributing to abstracts, manuscripts, presentations, and the funder’s biannual progress reports. Importantly, our level of engagement in each component was determined by us. We worked diligently with the team to represent the reality that was appropriate for all parties.

Our current experience is in contrast to several tokenistic patient partner experiences in the literature [11, 12] and some we also encountered on previous projects. For example, while it was frustrating and disrespectful that patient partners on an unrelated former project were not acknowledged in the final report (or even asked to contribute/review it in advance), we have been offered and supportively encouraged in this project to contribute to all forms of project outputs. In another example, a patient partner was told that the operational procedures of projects were not part of the patient partner role, whereas we have been treated as full, active project team members in this project and have changed the way the project functions in many regards (e.g., standing meeting agenda item, circulating PowerPoint slides in advance, online document repository, etc.). Our previous experiences helped us to be more selective in which projects we were willing to join, and the current project really supported us to find and use our voice as patient partners. It has even given us the confidence to carry our voice and apply these lessons learned into additional projects we have since joined.

## Conclusions

During the entire research project, the team learned from these lessons. The team carved out and created a safe place, free from stigma, where all were treated equally. Our oral and written presentations on patient engagement contributed to an exponential growth in confidence about the meaningfulness of the research; we felt our truth accepted, and our emotional labour recognized and appreciated. These experiences fostered a shared understanding, which positively impacted team development and cohesion. Researchers reported greater contextual understanding, unique insights, and more meaningful research through patient partner contributions. Co-learning of this kind is rare in mental health research.

We look forward to future iterations of the study and to the publications and presentations. These will showcase how researchers and patient partners with lived experience can successfully work together for the common goal of meaningful mental health care research. We believe that the lived experience of patients can and should go hand-in-hand with research. This helps to ensure that the research findings reflect the actual experiences, knowledge and expertise of those with lived experience. Successful engagement acknowledges and celebrates the patient partner experience. We hope that this article will be a useful guide to other patients and researchers as they contemplate and then begin their journey of co-learning together.

## Data Availability

Not applicable.
